# Dietary omega-3 polyunsaturated fatty acids interact with select gut microbes to produce bioactive metabolites found in infant gut microbiomes

**DOI:** 10.64898/2026.10.10.756005

**Published:** 2026-10-12

**Authors:** Janine M.C. Comrie, Xiaoqing Tan, Henry H. Le, Min-Ting Lee, Annett Richter, Sharon C. Thompson, Kevin R. Besler, Elizabeth L. Johnson

## Abstract

Gut microbes metabolize nutrients in infant diets, producing compounds that play roles in host health. Long-chain polyunsaturated fatty acids (LCPUFAs) are an important component of infant diets, supporting neurological development and immune regulation. Emerging evidence demonstrates that gut microbes, alongside the host, participate in the metabolism of dietary lipids, highlighting an underexplored avenue for LCPUFA biology. In this work, alkyne-tagged eicosapentaenoic acid (EPA ^Alk^ ) was used to investigate interactions between LCPUFAs and the gut microbiota. Flow cytometry and comparative metabolomics identified gut microbial EPA interactors in the ileum of mice. Further comparative metabolomics analysis identified the conversion of EPA to *N* -acyl amino acids, including attachment to phenylalanine (EP-Phe) and leucine (EP-Leu). Targeted analysis of *ex vivo* stool cultures determined that EP-Phe and EP-Leu are produced by gut bacteria. A survey of lipid-relevant infant gut bacteria determined various flow cytometry-confirmed EPA-microbe interactions and identified *Enterococcus faecalis, Bacteroides thetaiotaomicron,* and *Clostridium bifermentans* as competent producers of EP-Phe or EP-Leu. Thus, both novel metabolites from diet-derived inputs and microbial producers of these metabolites were identified. Together, this highlights important microbiome-centric consequences of LCPUFA consumption and emphasizes that the gut microbiome may be an important consideration in targeted supplementation strategies.

